# Genome-wide analysis of *UGT* gene family identified key gene for the biosynthesis of bioactive flavonol glycosides in *Epimedium pubescens* Maxim.

**DOI:** 10.1016/j.synbio.2022.07.003

**Published:** 2022-07-31

**Authors:** Yu Yao, Jiajun Gu, Yanjiao Luo, Yuanyue Wang, Yongzhen Pang, Guoan Shen, Baolin Guo

**Affiliations:** a Key Laboratory of Bioactive Substances and Resources Utilization of Chinese Herbal Medicines, Ministry of Education, Institute of Medicinal Plant Development, Chinese Academy of Medical Sciences, Peking Union Medical College, Beijing, 100193, China; b Institute of Animal Science, Chinese Academy of Agricultural Sciences, Beijing, 100193, China

**Keywords:** *Epimedium pubescens* Maxim., Flavonoids, UDP-glycosyltransferase, Phylogenetic analysis, *EpGT60*

## Abstract

*Epimedium pubescens* Maxim. is a well-known traditional Chinese medicinal herb with flavonol glycosides as the major pharmaceutically active compounds. UDP-glycosyltransferases (UGTs) are a group of enzymes responsible for the glycosylation of flavonoid glycosides. In this study, a genome-wide analysis was performed to identify *UGT* family genes in *E. pubescens*. As a result, a total of 339 putative *UGT* genes were identified, which represents the largest *UGT* gene family known thus far, implying a significant expansion of the *UGT* gene family in *E*. *pubescens*. All *EpUGTs* were unevenly distributed across six chromosomes, and they were classified into 17 major groups. The expression profiles showed that *UGT* genes were differentially expressed in roots, leaves, flowers, shoots and fruits. In particular, several *EpUGTs* were highly induced by high light intensity, which was consistent with the accumulation level of bioactive flavonoids in *E*. *pubescens*. Six *UGT79* genes that were preferentially expressed in roots or leaves were successfully expressed in *E. coli*, and only the recombinant EpGT60 protein was found to be active toward 8-prenylkaempferol and icaritin to produce the key bioactive compounds baohuoside II and baohuoside I. The optimal temperature, pH, *k*_*m*_ and *V*_*max*_ were determined for the recombinant EpGT60 protein. In addition, expression of recombinant EpGT60 in *E. coli* cell culture led to successful production of baohuoside II when fed 8-prenylkaempferol. Our study provides a foundation for further functional characterization of *UGT* genes in *E. pubescens* and provides key candidate genes for bioengineering bioactive flavonoids in *E. pubescens.*

## 1 Introduction

With 68 species, *Epimedium* is the largest herbaceous genus in the Berberidaceae family. For over two thousand years, more than 15 species in this genus have been widely used as traditional Chinese medicines (TCMs) [1]. The leaves of *Epimedium* have many beneficial effects on human and animal health as tonics, antirheumatics, antiosteoporotics, and aphrodisiacs in curing sexual dysfunction [2]. Flavonoids have been proven to be the major bioactive components in the leaves of *Epimedium*, which are mainly composed of prenylated flavonol glycosides, such as epimedin A, epimedin B, epimedin C, and icariin [[2], [3], [4]]. The stability and solubility of flavonols could be largely improved by glycosylation due to obvious changes in physicochemical properties, which greatly affects the accumulation of flavonol glycosides in plant cells [5,6]. Furthermore, glycosylation is the key and last modification for the biosynthesis of flavonol glycosides in *Epimedium.* However, the glycosylation mechanism of flavonol glycosides in *Epimedium* remains largely unclear.

Glycosyltransferase (GT, EC 2.4.x.y) is responsible for the transfer of glycosyl moieties from activated donor molecules to receptor molecules (such as sugars, lipids, nucleic acids, proteins, antibiotics, and other small molecules) to form a variety of glycoside compounds [7]. According to the most recent update of the CAZy system (http://www.cazy.org/Glycosyl-Transferases.html), GTs from different species can be classified into 115 families based on their amino acid sequences, catalytic mechanisms, and conserved sequence motifs. Family-1 glycosyltransferases (GT1), also known as UDP-dependent glycosyltransferases (UGTs), are the most common glycosyltransferases in the plant kingdom [7,8] and can transfer glycosyl moieties from UDP sugars to a wide range of acceptors, such as flavonoids, terpenes, auxin, cytokinin, salicylic acid, and many other compounds. These glycosylated compounds play important roles in plant growth, development, disease resistance, and interaction with the environment [5,6].

UDP-glycosyltransferase genes represent a large and diverse gene family in various plant species. Over 100 *UGTs* have been identified from *Arabidopsis thaliana*, which can be clustered into 14 groups based on amino acid sequences [7]. *Quercus suber* has 312 UGT family members, which is the largest UGT family known thus far [9]. A large number of *UGTs* have also been identified in many other plant species, including *Vitis vinifera*, *Malus × domestica*, *Citrus grandis*, *Sorghum bicolor*, and *Prunus persica*, etc [[9], [10], [11], [12]]. Apart from crops and model plants, many UGTs are involved in the modification of the bioactive components in medicinal plants, including flavonoid glycosides, terpenoid glycosides, etc [13]. A total of 124 UGTs were identified from genome of *Scutellaria baicalensis*, among which 6 glucosyltransferases (SbUGTs) convert baicalein to oroxin A (baicalein 7-O-glucoside) and 4 glucuronosyltransferases (SbUGATs) catalyzed baicalein to baicalin in vitro enzyme [14]. UGTPg1 was isolated from ginseng (*Panax ginseng*), it could glycosylate the C20–OH of protopanaxadiol (PPD) and protopanaxatriol (PPT), produce bioactive ginsenosides, which were the main pharmacologically active natural compounds in *P. ginseng* [15]. Glycosylation directly or indirectly affects the medicinal activity by optimizing the properties of pharmaceutical compounds [5]. To date, a few UGTs have been proven to be involved in the biosynthesis of icariin, one of the most important active ingredients of flavonol glycosides in *Epimedium* [[16], [17], [18], [19]]. However, the function of hundreds of *UGT* gene members remains unclear. Therefore, a comprehensive analysis of all *UGT* genes will be important for deciphering the complete mechanism of glycosylation related to the biosynthesis of flavonol glycoside in genus *Epimedium*.

In this study, we identified 339 *UGT* genes with high-quality reference genome information for *E. pubescens* and classified them into 16 groups based on sequence alignment and phylogenetic analysis. The expression profiles of *UGT* genes in various tissues of *E. pubescens* were analyzed using RNA-seq. Specifically, a novel flavonoid *UGT* gene (named *EpGT60*) was revealed to encode a key UGT to catalyze the rhamnosylation of prenylflavonols at the 3-OH position in *in vitro* enzymatic assays. This study provides a basis for analyzing the functional characteristics of *UGT* genes of *Epimedium* and is beneficial for understanding the glycosylation mechanism of the main active components in *Epimedium*.

## 2 Materials and methods

### 2.1 Plant materials and growth conditions

Ninety 2-year-old healthy seedlings of *Epimedium pubescens* Maxim. were collected from Lei Shan County (16°N, 108°E) in Guizhou Province, China. The *E. pubescens* plants were identified by Professor Baolin Guo from the Institute of Medicinal Plant Development, Chinese Academy of Medical Science, Peking Union Medical College. The plants were transferred to plastic pots and placed in a greenhouse at the Institute of Medicinal Plant Development. The plants were randomly subjected to different light intensities: L1: 5.5 ± 2.5 μmol m^−2^·s^−1^, L2: 18.2 ± 2.5 μmol m^−2^·s^−1^ and L3: 90.9 ± 2.5 μmol m^−2^ s^−1^ for 16 h per day as previously reported [20].

### 2.2 Genome-wide identification of *UGT* genes in *E. pubescens*

To identify *UGT* genes in *E. pubescens*, the functions of known UGTs from other plants were first used as a query to search against the genome of *E. pubescens* with the BLASTp algorithm. Furthermore, the *UGT* candidate genes were identified through a conservative domain search in the Pfam database (http://pfam.xfam.org/) with Pfam accession number PF00201. The results were combined to remove abundant sequences. The conserved domains of the plant secondary product glycosyltransferase box (PSPG box) were further confirmed by NCBI CDD (http://www.omicsclass.com/article/310).

The online CELLO v2.5 system (http://cello.life.nctu.edu.tw/cello.html) was used to predict the subcellular localization [21]. The molecular weight (MW) and isoelectric point (PI) were analyzed using the online ExPASy program (http://web.expasy.org/compute_pi/) [22].

### 2.3 Sequence alignment and phylogenetic analysis

Multiple sequence alignments of *E. pubescens* UGT protein sequences were aligned with the ClustalX v2.0 program [23]. The phylogenetic tree was constructed with MEGA X software based on the UGT protein sequences through the neighbor-joining (NJ) method with a bootstrap value of 1000 [24].

### 2.4 Analysis of chromosomal locations and gene duplication

The chromosomal distribution of *UGT* genes was visualized with the TBtools program (https://github.com/CJ-Chen/TBtools) [25]. Multiple Collinearity Scan Toolkit (MCScanX) with default parameters was used to analyze the tandem repeats and segmental duplication events of the *UGT* gene family in the *E. pubescens* genome [26]. Ka and Ks values were calculated using the TBtools program (https://github.com/CJ-Chen/TBtools) [25].

### 2.5 Analysis of gene structure and conserved motifs

To identify conserved motifs, the UGT protein sequences were analyzed using the MEME online program (http://meme-suite.org/) [27] with the default parameters, except for the following parameter: the maximum number of motifs = 15. The exon–intron structures of UGTs were drawn by using the TBtools program (https://github.com/CJ-Chen/TBtools) [25].

### 2.6 Analysis of the expression profile of *EpUGT* genes

The expression profiles of *EpUGT* genes in various organs and developmental stages were determined using high-throughput RNA sequencing from 5 tissues (roots, shoots, leaves, flowers, and fruits) of *E. pubescens*. The expression patterns of *UGT* genes in response to different light intensities were also analyzed by using RNA-seq in *E. pseudowushanense* [20]. Heatmaps of the expression levels of these *UGT* genes were drawn by using the TBtools program (https://github.com/CJ-Chen/TBtools) [25].

### 2.7 Heterogeneous expression of *UGT* in *E. coli*

An Eastep® Super total RNA Extraction Kit (Promega, Shanghai, China) was used to extract total RNA from fresh leaves of *E. pubescens*, and then the total RNA was reverse transcribed into complementary DNA (cDNA) by a FastKing One-Step RT–PCR Kit (TIANGEN Biotech, Beijing, China). Nested PCR amplification was performed using Q5® High-Fidelity DNA Polymerases (New England BioLabs, MA, USA) with cDNA from leaves of *E. pubescens* and gene-specific primers (Supplementary Table 1). PCR products were purified using an AxyPrep DNA Gel Extraction Kit (Corning, NY, USA), amplified by specific primers with the sites for restriction digestion, and then cloned into the pMAL-c2X vector (New England Biolabs, MA, USA) using a LanGene Seamless Cloning Assembly Kit (LANY, Beijing, China). The recombinant pMAL-c2X-EpUGT vectors were sequenced and transformed into *E. coli* strain BL21 (DE3) competent cells (TransGen Biotech, China).

The recombinant BL21 (DE3)/pMALc2X-EpGTs were incubated at 37 °C until the OD_600_ reached 0.5. Then, 0.45 mM isopropyl-β-d-thiogalactopyranoside (IPTG) was used to induce the recombinant protein at 16 °C for 23 h with a shaking speed of 100 rpm. The bacterial cells were collected by centrifugation at 4 °C and affinity-purified to obtain the maltose-binding protein (MBP) fusion proteins according to the pMAL™ Protein Fusion and Purification System (New England Biolabs, MA, USA).

### 2.8 Enzymatic assays of recombinant EpGT60 protein

The six recombinant EpGT proteins (∼5 μg) were incubated at 30 °C for 1 h with 100 mM Tris-HCl (pH 7.5), 1 mM DTT, 4 mM UDP-glucose/UDP-rhamnose/UDP-xylose and 0.5 mM substrates for a final volume of 50 μL. Authentic compound standards were purchased from Shanghai Yuanye Bio-Technology Co., Ltd. (Shanghai, China). Reactions were terminated after 1 h by the addition of 100 μL of ice-cold MeOH, and the products were separated by centrifugation at 14,000 rpm for 10 min.

The reaction products were detected using ACQUITY UPLC (UPLC I-class; Waters, Milford, MA, USA) with an ACQUITY UPLC BEH C18 column (2.1 × 100 mm, 1.7 μm, Waters, Milford, MA, USA) at 35 °C. Water (A) and acetonitrile (B) were used as the mobile phase. The elution gradient was achieved as follows: 0–5 min (5%–40% B), 5–15 min (40%–100% B), 15–16 min (100% B), and 16–17 min (5% B). The UPLC analysis was run at a flow rate of 0.3 mL min^−1^ and detected at a wavelength of 270 nm.

LC–MS/MS analysis was performed on a Waters ACQUITY UPLC I-Class/Xevo G2-XS QTOF (Waters, Milford, MA, USA) with a ZORBAX Eclipse Plus C18 (3.0 mm × 155 mm, 1.8 μm) at 30 °C. Water (A) and acetonitrile (B) were used as the mobile phases. The elution gradient was achieved as follows: 0–1 min (5% B), 1–8 min (5%–30% B), 8–12 min (30%–40% B), 12–16 min (40%–95% B), 16–17 min (95%–100% B), 17–21 min (100% B), 21–21.2 min (100%–5% B), and 21.2–25 min (5% B). The operating conditions were as follows: flow rate of 0.4 mL min^−1^ with positive ion ESI mode, capillary voltage of 2.5 kV, cone voltage of 25 V, and desolvation gas flow of 1000 L/h. The mass-to-charge ratio was scanned from 200 to 1,500 *m/z*.

### 2.9 Enzymatic kinetics of the recombinant EpGT60 protein

Enzymatic assays with UDP-rhamnose as the donor and 8-prenylkaempferol as the acceptor were utilized. To study the optimal reaction temperature, the reaction mixtures were incubated at different temperatures from 20 °C to 50 °C. For the optimal pH, the enzymatic reaction was performed in various reaction buffers with pH values in the range of 4.5–9.0 (Tris-HCl buffer) and 9.0–10.5 (Na_2_CO_3_–NaHCO_3_ buffer). For the optimal incubation time, the reaction mixture was incubated at 30 °C and then collected at given time points from 1 h to 36 h. The enzymatic reaction was performed with 5 μg recombinant EpGT60 in a final volume of 50 μL at the optimal temperature, pH, and incubation time. The reaction was terminated by the addition of an equal volume of ice-cold MeOH and then detected by UPLC analysis.

For kinetic analysis, purified recombinant EpGT60 proteins (∼5 μg) were added to reaction mixtures containing 1 mM DTT, 50 mM Tris-HCl (pH 7.0), and 4 mM UDP-rhamnose in a final volume of 50 μL. The concentrations of flavonoid substrates were 100 μM, 200 μM, 300 μM, and 500 μM. Reactions were stopped by the addition of 50 μL ice cold MeOH after 1 h of incubation at 30 °C. After centrifugation at 14,000 rpm for 10 min, the reaction products were analyzed by UPLC. The kinetic parameters *K*_*m*_ and *V*_*max*_ were calculated by using the GraphPad Prism version 7.0.0 software for Windows (GraphPad Software, San Diego, California, USA).

### 2.10 Overproduction of baohuoside II from 8-prenylkaempferol in *E. coli*

BL21 (DE3)/pMALc2X-EpGT60 cells were cultured until the OD_600_ reached 0.6–0.8. IPTG (0.45 mM) was used to induce protein expression at 16 °C for 24 h. The bacterial cells were collected by centrifugation at 4,000 rpm for 10 min and suspended in 50 mL of M9Y medium (containing 0.025% yeast extract and 0.025% α-l-rhamnopyranose). 8-prenylkaempferol at a final concentration of 200 μM was added to the culture medium, followed by incubation at 30 °C for 48 h. The cell cultures were extracted with ethyl acetate and then evaporated to dryness. The dried samples were dissolved in 200 μL MeOH for UPLC analyses.

### 2.11 Three-dimensional modeling of EpUGTs

The three-dimensional models of the EpUGTs were built with the SWISS-MODEL server [28] at http://swissmodel.expasy.org. and docked with 8-prenylkaempferol (compound CID: 5318624) and UDP-rhamnose (compound CID: 192751) as the sugar donor using Maestro software (Schrödinger, LLC, New York, NY). The models were visualized using the PyMOL molecular graphics system (http://www. pymol.org).

## 3 Result

### 3.1 Genome-wide identification of *UGT* genes in *E. pubescens*

The C-terminal sequence of plant UGTs contain a unique and well-conserved region of 44 amino acids called the plant secondary product glycosyltransferase (PSPG) box. The PSPG box has been found in all known plant UGTs and represents the nucleotide-diphosphate-sugar binding site [7]. By analyzing the *E. pubescens* genome sequences generated by our group, we identified 339 putative *UGT* genes in the genome. They were all confirmed by the presence of a conserved PSPG box. The deduced protein length of EpUGTs ranged from 304 to 528 amino acids. The theoretical isoelectric point (PI) ranged from 4.76 to 7.74 (average PI = 5.65), and the molecular weight (MW) varied from 33.98 kDa to 59.02 kDa (average MW = 51.04 kDa) (Supplementary Table 2). The theoretical cellular localization analysis showed that most EpUGT proteins (77.29%) were predicted to be localized in the cytoplasm, followed by the inner membrane (9.44%), outer membrane (7.96%), and periplasm (5.01%), and only one protein was predicted to localize in the mitochondria (Supplementary Table 2).

### 3.2 Chromosomal distribution and synteny analysis of *UGT* genes

**Fig. 1:**
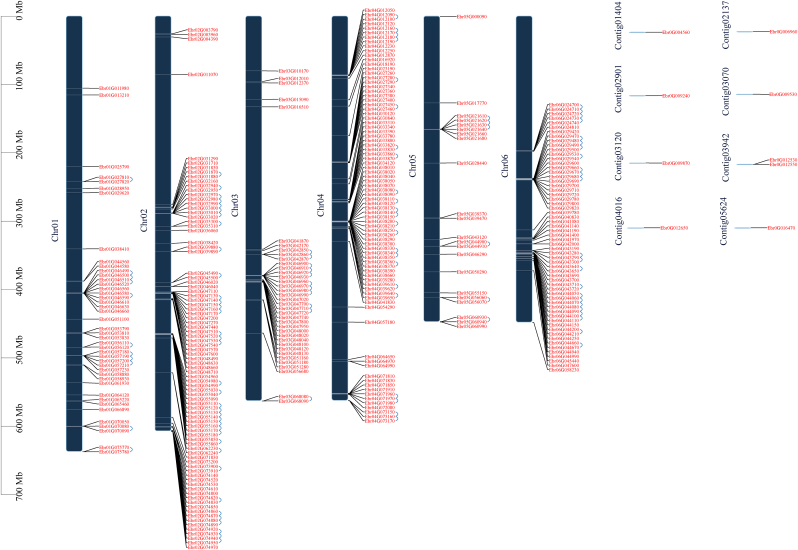
**Chromosomal locations and tandem duplication of *UGT* family in *E. pubescens*.** The blue lines indicate tandem duplication events. The chromosome name is indicated on the left side of each chromosome.

To provide an overview of the distribution of all *UGT* genes, the location of *UGT* genes on chromosomes was further investigated based on our current genome information of *E. pubescens*. Among them, 331 *UGT* genes were generally unevenly distributed across six chromosomes, while eight *UGT* genes were mapped to contigs. Specifically, *UGT* genes were preferentially located on Chr02 (97), followed by Chr04 with 83 genes, and Chr05 had the fewest genes, with 22 (Fig. 1).

The duplication events of *UGT* genes were further investigated in *E. pubescens*. Eventually, 110 gene pairs were considered to originate from tandem duplication events, and they were unequally distributed on all 6 chromosomes. Specifically, Chr03, Chr04, and Chr06 had more than eight clusters, indicating hot spots for *UGT* gene duplication, which also explained why more *UGT* genes were located on these chromosomes. In addition to the tandem duplication events, 2 segmental duplication events involving 4 *UGT* genes were also identified (Supplementary Table 3 and Fig. 1). These results suggested that many *UGT* genes possibly originated from tandem duplication events, which might act as one of the main driving forces of UGT evolution.

To better understand the evolutionary constraints on the *UGT* gene family, the nonsynonymous (Ka) and synonymous (Ks) substitution rates between these duplicated gene pairs were calculated (Supplementary Table 3). All duplicated UGT gene pairs had Ka/Ks values of <1, suggesting that the *E. pubescens UGT* gene family might have experienced strong purifying selective pressure during its evolution. Moreover, the approximate date of duplication events was calculated using T = Ks/2λ, assuming that the synonymous substitutions per site per year were 6.98 × 10^−9^ for Ranunculales [29]. The tandem duplications of *UGT* genes were estimated to have originated 40.11 million years ago (Mya) (Ks = 0.56), while the segmental duplications might have originated 70.91 Mya (Ks = 0.99) in *E. pubescens*.

### 3.3 Phylogenetic analysis of the *UGT* gene family in *E. pubescens*

**Fig. 2:**
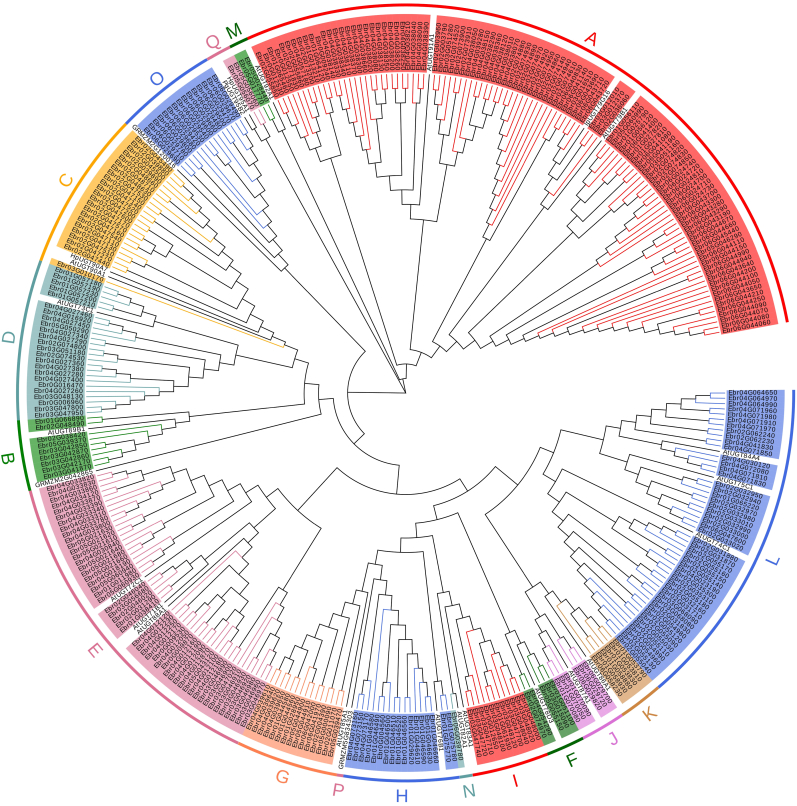
**Phylogenetic analysis of UGT family genes in *E. pubescens*.** The phylogenetic tree was constructed by using the full-length sequences of 339 *Epimedium* UGTs and 32 UGTs from *Arabidopsis thaliana*, *Zea mays*, *Punica granatum* and *Hieracium pilosella*. 17 groups (A–Q) are indicated in different color.

A phylogenetic tree was constructed by aligning the full-length amino acid sequences of *E. pubescens* UGTs with 26 UGTs from *Arabidopsis* [30] and 6 UGTs from other plants (Supplementary Table 4). The *E. pubescens* UGTs were clustered into 16 major groups (Fig. 2), including 14 UGT groups (A-N) described initially in *Arabidopsis* that were considered conserved UGT groups [12] and 2 O and Q groups containing 15 and 2 UGT members, respectively. The number of UGTs varied largely in each group. Group A was the largest group (105), followed by Group E (49) and Group L (49). Group N contained only 1 member (Fig. 2).

The *UGT* genes within the same group were often closely linked together, but they also occurred in different locations on the chromosomes. All 105 *UGT* genes within the A group were unevenly located on all six chromosomes and were mainly located on Chr06 and Chr04. Nine genes of the B group were found on Chr01, Chr02, Chr03, and Chr05. The 24 genes of the D group were randomly distributed across five chromosomes (Chr 01–05). All five genes in the F group were located similarly to those in the D group.

In *A. thaliana*, UGT79B2 and UGT79B3 in Group A were annotated as flavonol 3-*O*-glucosyltransferases, UGT79B1 in Group A converted cyanidin 3-O-glucoside to cyanidin 3-O-xylosyl(1 → 2)glucoside [31,32], and UGT73C6 in Group D was annotated as a flavonol 7-*O*-glucosyltransferase [33]. In addition, some AtUGTs responsible for glycosylation of flavonols and anthocyanins were found in Groups B and F [33]. Therefore, Groups A, B, D and F were most likely involved in the glycosylation of prenylflavonols in *E. pubescens*.

### 3.4 Analyses of conserved motifs of UGTs

**Fig. 3:**
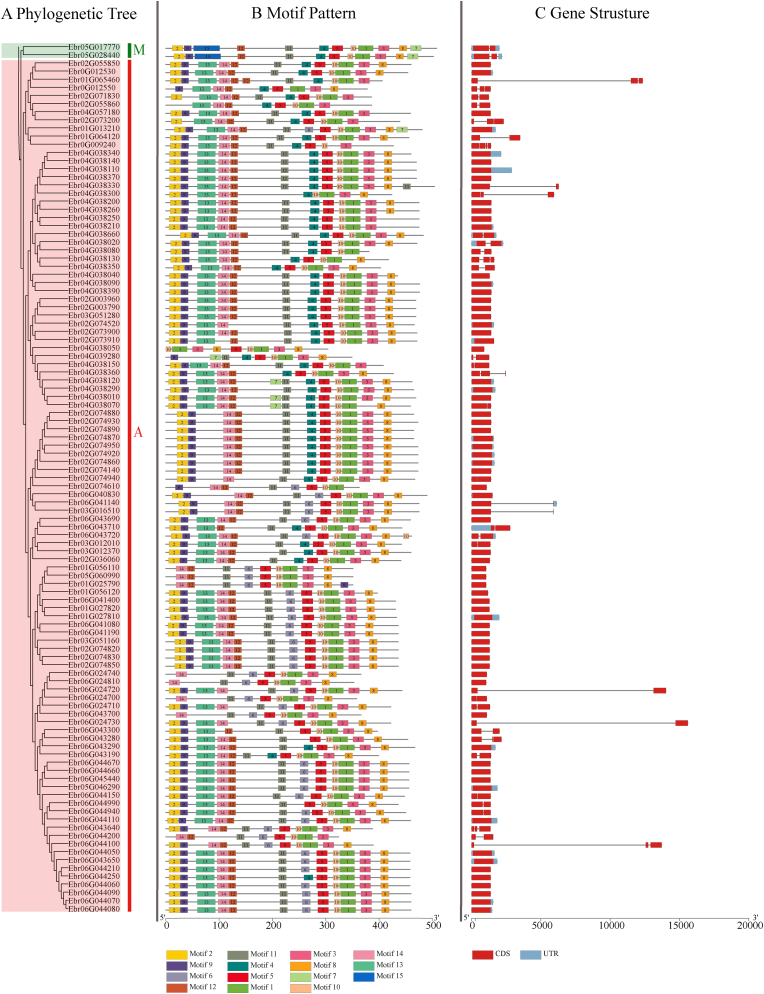
**Phylogenetic relationships, protein motifs and gene structures of UGTs from the group A and M**. (A) The phylogenetic tree was constructed based on UGT proteins from A and M groups, which were indicated in red and green, respectively. (B) The motif composition of UGT proteins. Numbers 1–15 of motifs are shown in different colored boxes. (C) Exon-intron structure of *UGTs*. Red boxes indicate exons, blue boxes indicate UTR, black lines indicate introns.

To further determine the conserved domain of UGT family genes, 15 conservative motifs were identified through the MEME web server (Fig. 3, Supplementary Figs. 1 and 2). Motifs 1 and 3 were found in the PSPG domain (WAPQ-VL-H-AVG-FLTHCGWN-STLES-GVP-WPM-DQ) with minor variations. These results showed that members within the same group had very similar or identical conserved motifs. The position of motifs was relatively constant with an order of motif 2-9-12-11-6-5-10-1-3-8 in most UGTs. Motifs 13 and 14 were unique to the A and M groups, which may be important for these UGT proteins to catalyze flavonols or anthocyanins, while motif 15 was specific to the C and D groups. Most members in Group A lacked motif 7 at the tail, which is common to all other groups (Fig. 3, Supplementary Figs. 1 and 2). These particular conserved motifs potentially explained the functional differences among UGT family members. In general, the reliability of the present group categorization was strongly supported by the conserved motif composition, gene structure, and phylogenetic analysis (Fig. 3, Supplementary Figs. 1 and 2).

### 3.5 Tissue-specific expression of *UGTs* in *E. pubescens*

**Fig. 4:**
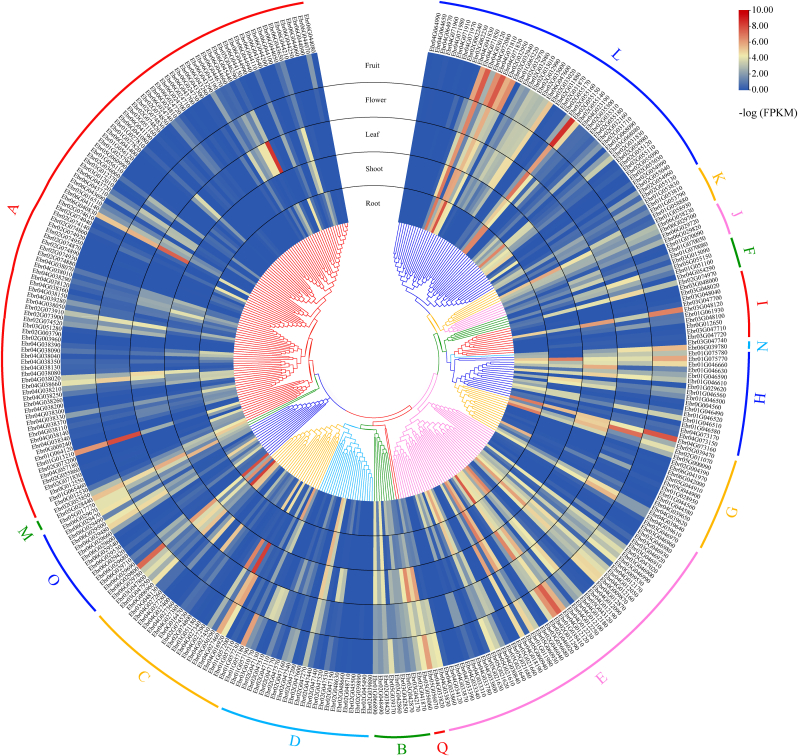
**Expression levels of all *UGTs* in various tissues by RNA-seq.** The genes were classified into 16 different groups with different color based on phylogenetic tree.

An RNA-seq analysis was carried out to determine the expression pattern of 339 *UGT* genes in roots, shoots, leaves, flowers and fruits of *E. pubescens*. A total of 40 (11.80%), 37 (10.91%), and 31 (9.14%) UGTs showed relatively high transcript levels in roots, leaves, and fruits, respectively (Fig. 4 and Supplementary Table 5). In particular, *Ebr02G055150*, *Ebr04G071830*, and *Ebr04G030120* in Group L showed high expression levels in all five tissues (Fig. 4 and Supplementary Table 5), indicating that these UGTs may be involved in a broad spectrum of biological processes during growth and development.

For the genes in Groups A, B, D, and F that may use flavonoids as substrates, UGTs in Groups A and B were predominantly expressed in leaves, UGTs in Group D were abundantly expressed in roots and leaves, and UGTs in Group F showed high expression levels in leaves and flowers (Fig. 4).

### 3.6 Expression levels of *UGT* genes in response to different light intensities in *E. pubescens*

**Fig. 5:**
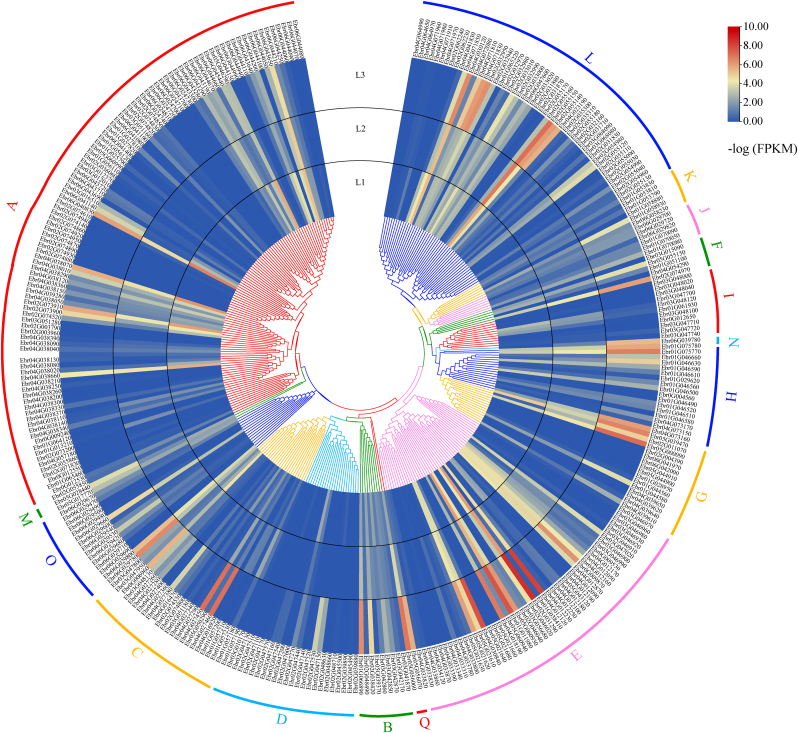
**Expression levels for *Epimedium UGTs* in response to different light intensities.** The genes were classified into 16 different groups with different color based on phylogenetic tree.

In a previous study, it was proven that flavonoid contents from *E. pseudowushanense* varied under different light intensities, and a large amount of icariin was produced under high light intensity [20]. To test whether the expression levels of *UGT* genes were altered under different light intensities, transcript profiles were investigated in the leaves of *E. pseudowushanense* under different light intensity levels (Fig. 5 and Supplementary Table 6). After 30 days of treatment under different light intensities, the transcript levels of *Ebr06G041140* and *Ebr02G073900* from Group A, *Ebr01G066890* and *Ebr02G038420* from Group B, *Ebr02G047530* from Group C, and *Ebr05G055150* and *Ebr02G074970* from Group F were induced under high light intensity, while *Ebr04G027460*, *Ebr04G027450*, and *Ebr02G031290* from Group D were highly expressed under high light intensity. More than ten genes from both the E and L groups were significantly induced by high light intensity. A similar pattern was also observed for three members from Groups G, H and O, and two genes from Group Q (Fig. 5 and Supplementary Table 6).

### 3.7 Functional characterization of the UGT79 family

Previous studies showed that UGTs with prenylflavonoids as substrates were mainly from Group A or Q [17,18]. Among Group A, the UGT79 family contains large amounts of GGTs that are able to attach additional sugar moieties to the existing sugar group of flavonoid glycosides. Among them, 59 *UGTs* (EpGT1-59) were unevenly located on different chromosomes, and another *UGT* (EpUGT60) was not anchored to any chromosome. RNA-seq data showed that these genes had tissue-specific expression patterns (Supplementary Fig. 3). Twenty-two of them were preferentially expressed in either roots or leaves (more than 2-fold higher than in fruits) (Supplementary Table 7); thus, they were cloned with gene-specific primers. However, only seven of them were successfully cloned and expressed in *E. coli*, namely, *EpGT21*, *EpGT36*, *EpGT42*, *EpGT44*, *EpGT50*, *EpGT56* and *EpGT60* (Supplementary Table 7).

**Fig. 6:**
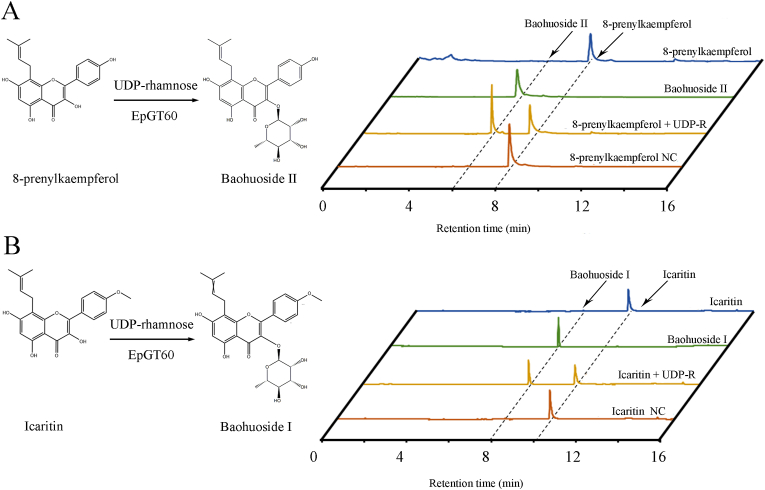
**Enzymatic activity of recombinant EpGT60 protein.** (A) Verification of the products from the reaction catalyzed by recombinant EpGT60 protein using 8-prenylkaempferol as substrate. (B) Verification of the products from the reaction catalyzed by recombinant EpGT60 protein using icaritin as substrate. Left: the reaction catalyzed by EpGT60. Right: UPLC chromatographs. NC was negative control reaction with boiled protein.

The purified recombinant proteins were then incubated with 23 different flavonoid compounds (Table 1) and UDP-glucose/UDP-rhamnose/UDP-xylose. However, only the recombinant EpGT60 protein (Supplementary Fig. 4) showed enzymatic activity toward 8-prenylkaempferol and icaritin with UDP-rhamnose as the donor, and the other six recombinant proteins did not show any activity against these substrates (Fig. 6 and Supplementary Fig. 5). Using boiled protein as a negative control reaction (NC), the enzyme reaction of EpGT60 with 8-prenylkaempferol as a substrate produced baohuoside II (Fig. 6A) and with icaritin as a substrate produced baohuoside I (Fig. 6B). Both the retention time and LC-MS spectra of enzymatic products were the same as baohuoside II and baohuoside I authentic standards, with monoisotopic *m/z* values of 500.1199 and 514.1780, respectively (Fig. 6, Supplementary Fig. 6).

**Table 1:** Flavonoids used in the enzymatic assays with EpGT60.

Substrate type	Substrate names	EpGT60
**8-prenylflavonol**	8-prenylkaempferol	**+**
	Icaritin	**+**
	Baohuoside I	**-**
	Icariside I	**-**
	Icariin	**-**
**Flavonol**	Kaempferol	**-**
	Quercetin	**-**
	Kaempferide	**-**
	Galangin	**-**
	Tamarixetin	**-**
	Isorhamnetin	**-**
	Mearnsetin	**-**
**Flavanone**	Eriodictyol	**-**
	Naringenin	**-**
**Favonol glycosides**	Kaempferol-3-*O*-glucoside	**-**
	Quercetin-3-*O*-glucoside	**-**
	Isorhamnetin-3-*O*-glucoside	**-**
	Kaempferol-3-*O*-rhamnoside	**-**
	Quercetin-3-*O*-rhamnoside	**-**
	Isorhamnetin-3-*O*-rhamnoside	**-**
	Kaempferol-3-*O*-galactoside	**-**
	Quercetin-3-*O*-galactoside	**-**

In detail, the *EpGT60* gene has an ORF of 1422 bp, which encodes a protein of 473 amino acids with a calculated molecular mass of 52.1 kDa. The typical PSPG box, a highly conserved sequence of UGTs, was observed in the EpGT60 protein sequence (Supplementary Fig. 7). In addition, EpGT60 shared relatively high sequence similarity with other known UGTs with flavonoids as substrates, such as AtUGT79B1, IpUGT79G16, and PgUGT95B2 (Supplementary Fig. 7).

**Fig. 7:**
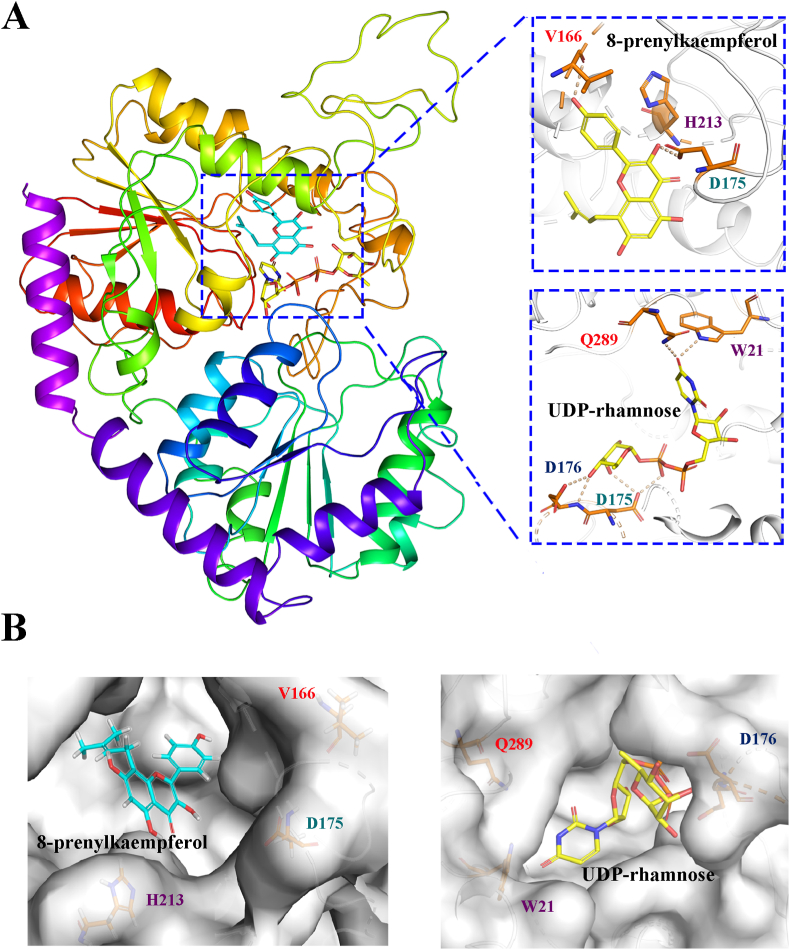
**Structure of EpGT60 protein.** (A) Structural overview of EpGT60 with 8-prenylkaempferol and UDP-rhamnose. (B) Substrate-binding pocket of EpGT60 with 8-prenylkaempferol was on the left, donor-binding pocket of EpGT60 with UDP-rhamnose was on the right.

Furthermore, the three-dimensional structures of EpGT21, EpGT36, EpGT42, EpGT44, EpGT50, EpGT56 and EpGT60 were analyzed by homology modelling and ligand docked with 8-prenylkaempferol and UDP-rhamnose (Fig. 7). The lower the docking score, the better the receptor binds to the enzyme. EpGT60 had the lowest docking score (−6.226) with 8-prenylkaempferol than other EpGTs, which might be the reason why only EpGT60 showed catalytic activity towards 8-prenylkaempferol (Supplementary Fig. 8 and Supplementary Table 8).

### 3.8 Enzymatic kinetics of the recombinant EpGT60 protein

**Fig. 8:**
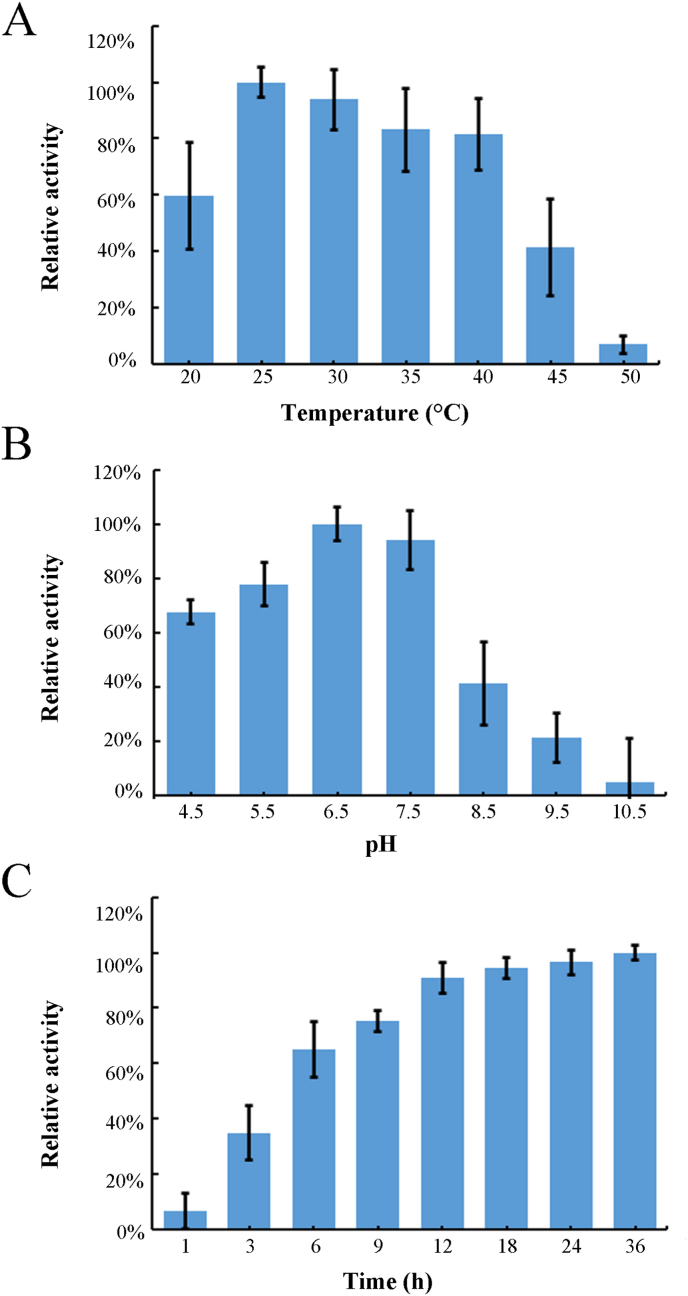
**Effects of temperature,** pH**, and incubation time on enzyme activity of EpGT60.** The enzymatic activity of EpGT60 were determined under different temperature (A), pH (B), and incubation time (C). UDP-rhamnose was used as a sugar donor and 8-prenylkaempferol was used as an aglycone acceptor. The values are presented as the means, and the error bars represent the SD (n = 3).

The catalytic properties of recombinant EpGT60 were further investigated under various temperature and pH conditions. The enzyme reaction of EpGT60 with 8-prenylkaempferol as a substrate showed maximal activity at 25 °C. When the temperature was increased above 40 °C, the enzyme activity decreased significantly (Fig. 8A). While the enzyme reactions were conducted under different pH conditions, the maximal activity of EpGT60 was observed at pH 6.5 (Fig. 8B). The relative conversion rate of substrates increased rapidly from 1 h to 12 h and then remained stable from 12 h to 36 h (Fig. 8C).

The kinetic parameters of EpGT60 were determined through a series of enzymatic assays with substrates of 8-prenyltkaempferol at various levels. The *K*_*m*_ value was calculated to be 288.6 μM, *V*_*max*_ value of 0.36 nmol min^−1^ and *K*_*cat*_ value of 62.99 s^−1^.

### 3.9 Production of baohuoside II in *E. coli* cell culture

Baohuoside II, glycosylated 8-prenylkaempferol, is a potential pharmaceutical agent for treating hepatic cancer. To test whether EpGT60 is able to produce baohuoside II in bacterial cells by a feeding assay, the bacterial strain containing pMAL-c2X*-*EpGT60 was cultured and fed 8-prenylkaempferol, and the products were detected by UPLC (Supplementary Fig. 9). The results showed that the recombinant bacterial cells carrying the *EpGT60* gene were able to produce 201.60 μg mL^−1^ of baohuoside II after 48 h of incubation, and the conversion rate of 8-prenylkaempferol was 53.32%. Therefore, it is a feasible alternative to produce baohuoside II on a large scale via bacterial cells.

## 4 Discussion

The structure of the flavonoid skeleton is relatively simple, and the huge diversity of flavonoids is largely attributed to hydroxylation, glycosylation, acylation, methylation, and other modifications [34]. Glycosylation is one of the key modifications in flavonoid biosynthesis, which may improve the solubility, stability, and bioactivity of flavonoids [35].

Glycosyltransferases with flavonoids as substrates have been identified in many important model plants and horticultural crops, such as petunia, *Arabidopsis*, grape, and soybean [[36], [37], [38], [39]]. Apart from crop and model plants, many UGTs are involved in the modification of bioactive components of flavonoids in medicinal plants. Glycosylation changes the physicochemical properties of natural products and thus directly or indirectly affects the efficacy of medicinal ingredients [13,40]. Therefore, it is essential to extend the study of UGTs in medicinal plants such as *E. pubescens*.

### 4.1 The *UGT* gene family is significantly expanded in *E. pubescens*

In the present study, a total of 339 *UGT* genes were identified in *E. pubescens*; thus, it is one of the largest UGT families in the plant kingdom known to date. The number of *UGT* genes in *E. pubescens* is even more than that in *Quercus suber,* with 312 UGT members, which was once thought to be the largest UGT family [9]. Such an expansion may be associated with the large genome size (3.14 Gb) of *E. pubescens*.

These *UGTs* were unevenly distributed across all chromosomes of the *E. pubescens* genome (Fig. 1). A similar distribution pattern of *UGTs* was also observed in other plants [10,11]. Gene duplication plays important roles in organismal evolution by generating novel genes that help to control physiological and morphological novelties. Tandem and segmental duplications are the main causes of gene family expansion in plants [41]. While 110 *UGT* gene pairs were identified as tandem duplications distributed on all 6 chromosomes (Supplementary Table 3 and Fig. 1), these genes are located close to each other on the chromosomes, suggesting that they may share the same function or act coordinately.

Both tandem repeat events and segmental repeat events are the main driving forces of UGT evolution in *E. pubescens*. The UGT gene family of *A. thaliana* contained 49 (44.95%) tandem repeats and 11 (10.09%) segmental duplications among a total of 109 *UGT* genes [42], which is much higher than that of *E. pubescens* (33.23% and 3.32%). The low ratio of tandem repeat events and segmental repeat events in *E. pubescens* is probably because whole-genome duplication increases *UGT* gene number across the *E. pubescens* genome, whereas local tandem duplication partially affects gene number in a genome due to duplication of restricted regions [43].

A phylogenetic analysis revealed 16 distinct groups (Fig. 2), including 14 highly conserved groups (A-N) found in *A. thaliana* [30] and two other groups, O and Q, that were observed in *E. pubescens*. The model plant *A. thaliana* lacks the O, P and Q groups [30], whereas *E. pubescens* lacks the P group. The large number of *UGT* genes in *E. pubescens* is primarily due to an expansion of Group A with 105 genes, which accounts for approximately 30.97% of the total *UGT* genes (Supplementary Table 2). It has been reported that the A group expanded faster than the other groups during the evolution of higher plants [12].

Flavonoids are usually glycosylated at both the 3-OH and 7-OH positions in *E. pubescens*. Generally, UGTs responsible for modifying the 3-OH position of flavonols are from the F group, whereas the UGTs glycosylating the 7-OH position are from the B, C or D groups [9]. However, it was found that UGTs catalyzing the 3-OH or 7-OH positions of prenylflavonol mainly came from group A in the *Epimedium* genus [17,18]. Considering that *Epimedium* is rich in various prenylflavonols, the A group, which is greatly expanded, may play an important role in glycosylation of prenylflavonols in the genus *Epimedium*.

Nevertheless, the G group contains only 13 members in *E. pubescens* (Supplementary Table 2). The members of the G group play important roles in the Rosaceae family [11]. It is worth noting that seven genes were found in Group F of *E. pubescens*, but only one or two genes of the F group were found in many higher plants [9,12]. However, more than five genes in the F group were found in flavonoid-enriched plants such as grapes, apples, and cottons [12,44], indicating the importance of the F group in flavonoid biosynthesis. It was also reported that UGTs in the F group were able to catalyze the glycosylation of flavonoids and anthocyanins [9]; therefore, *E. pubescens* might retain some original functions related to flavonoid glycosylation during evolution. In addition, Group N in *E. pubescens* contains only one member, which is consistent with previous reports in many other dicot plants, but the expansion of Group N was observed in monocots such as maize and rice [12], suggesting that they might be involved in monocot-specific glycosylation processes.

### 4.2 *UGT* gene family evolution under subfunctionalization in *E. pubescens*

It was found that all Ka/Ks ratios between duplicated *UGT* genes were less than 1 (Supplementary Table 3), suggesting that these genes experienced a strong purifying selective pressure. These results revealed that the maintenance of such a large UGT gene family is necessary for the survival of *E. pubescens.*

Plant UGTs do not contain a clear signal sequence, nor do they contain any known transmembrane or membrane guidance signals [11]. In addition, it has been proven that most UGTs show activity in the cytoplasm [45]. The theoretical cellular localization analysis showed that most *E. pubescens* UGTs (77.29%) were targeted to the cytoplasm, while some UGTs were possibly localized to the inner membrane, outer membrane, periplasm and mitochondria (Supplementary Table 2). The subcellular localization diversity of UGTs may result in diverse glycosylation of various substrates in different cell compartments [[46], [47], [48]]. In addition, flavonoid compounds are mainly synthesized in the cytoplasm, and the enzymes involved in flavonoid biosynthesis mainly exist in the cytoplasm near the endoplasmic reticulum as multienzyme complexes [49]. Therefore, UGTs located in the cytoplasm or endoplasmic reticulum are particularly noteworthy for the investigation of flavonoid glycosylation in future research.

Expression analysis by RNA-seq was conducted to better understand the roles of *UGT* genes in various tissues of *E. pubescens*. The expression of *UGT* genes in roots, leaves, and flowers was generally higher (Fig. 4 and Supplementary Table 5). The biosynthesis of secondary metabolites such as flavonoids is generally tissue specific. Significant functional divergence was found among different UGT groups, and duplicated genes usually exhibited diverse expression patterns. The differentiation of duplicated genes in expression is considered an important step in the functional differentiation to generate novel genes [50], which supported the hypothesis that UGTs were subfunctionalized during evolution. The different temporal and spatial expression patterns of duplicate genes may be closely related to the presence or absence of functionally important transcription factor-binding sites (TFBSs) in regulatory regions [51].

Furthermore, different light intensities significantly affected the transcript levels of many UGTs from Groups A, B, C, D and F in *E. pubescens* (Fig. 5 and Supplementary Table 6). Light was involved in the regulation of bioactive components in *E. pubescens*, and large amounts of flavonoid accumulation was induced under high light intensity treatment [20]. UGTs associated with flavonoid biosynthesis have been generally reported in Groups A, B, C, D and F, and they were significantly induced by high light intensity, indicating that they might play important roles in flavonoid glycosylation.

### 4.3 *EpGT60* is a novel rhamnosyltransferase toward prenylflavonols in *E. pubescens*

In the current study, *EpGT60* was found to be expressed in roots, shoots, leaves, flowers and fruits, with relatively higher levels in leaves and roots than in other tissues (Supplementary Fig. 3 and Supplementary Table 7), which was consistent with the predominant accumulation of icariin in leaves and roots of *E. pubescens*. The recombinant EpGT60 protein showed convincing activity to form 8-prenylkaempferol glycosides (Fig. 6 and Supplementary Fig. 6). Furthermore, EpGT60 could not catalyze the addition of glycosyl groups to kaempferol, suggesting that EpGT60 was different from the previously reported EpPF3RT from *E. pseudowushanense* or EkF3URhaT from *E*. *koreanum* [16,18]. These two recombinant enzymes have a similar function as EpGT60. However, EpGT60 can only recognize flavonols with a prenyl moiety at C-8, whereas both EpPF3RT and EkF3URhaT can also glycosylate kaempferol. The glycosylation mechanism of EpGT60 orthologs might have differentiated functionally in different *Epimedium* species, and the discrepant key amino acids may be the reason for their different catalytic functions (Supplementary Fig. 10). Although EpPF3RT, EkF3URhaT, and EpGT60 exhibited similar functions, their sequences varied distinctively in different species within the *Epimedium* genus, and whether their activities are dependent on these species-specific amino acids is worth further investigation.

Furthermore, we produced baohuoside II via bacterial cell culture with the recombinant EpGT60 protein, which is an important drug with anti-inflammatory activity [52]. Whole-cell biocatalysis reduces the steps for recombinant protein purification and expensive UDP-rhamnose. Due to the low content of these natural products and scarcity of germplasm resources of the *Epimedium* genus, it will be a great option to obtain bioactive flavonoids such as baohuoside II other than from *Epimedium* plants by using synthetic biology approaches.

## 5 Conclusions

In this study, 339 *UGT* genes were identified from the genome of *E. pubescens*, and the gene structures, phylogenetic relationships, gene duplications and expression profiles of *UGT* genes were investigated. In conclusion, the first investigation of *the UGT* gene family and a newly discovered *EpGT6*0 that was able to catalyze 8-prenylkaempferol provided powerful support for further studies of the functions of *UGT* in *E. pubescens*, as well as the biosynthesis and regulation of bioactive flavonoids in *E. pubescens*.

## Data availability

The genome database of *E. pubescens* is available at the National Genomics Data Center (NGDC, https://bigd.big.ac.cn/) under BioProject PRJCA006303. The raw data were uploaded to National Center for Biotechnology Information (NCBI) under project PRJNA747870.

## CRediT authorship contribution statement

**Yu Yao:** Methodology, Formal analysis, Visualization, Data curation, Writing – original draft. **Jiajun Gu:** Software, Visualization, Data curation. **Yanjiao Luo:** Investigation. **Yuanyue Wang:** Investigation. **Yongzhen Pang:** Writing – review & editing, Supervision. **Guoan Shen:** Conceptualization, Writing – review & editing, Supervision, Project administration. **Baolin Guo:** Conceptualization, Writing – review & editing, Supervision, Project administration.

## Declaration of competing interest

All authors disclosed no relevant relationships.

## Appendix A Supplementary data

Supplementary Information includes two sections: Supplementary Figure 1-10 and Supplementary Table 1-8.
