# Supplementary material for: Genome-wide analysis of UGT gene family identified key gene for the biosynthesis of bioactive flavonol glycosides in Epimedium pubescens Maxim
Source: Synth Syst Biotechnol. 2022 Jul 31;7(4):1095–107. doi: 10.1016/j.synbio.2022.07.003 (PMC9372747; doi:10.1016/j.synbio.2022.07.003)
Supplement: Multimedia component 1 [file mmc1.docx]

**Supplementary Figures**


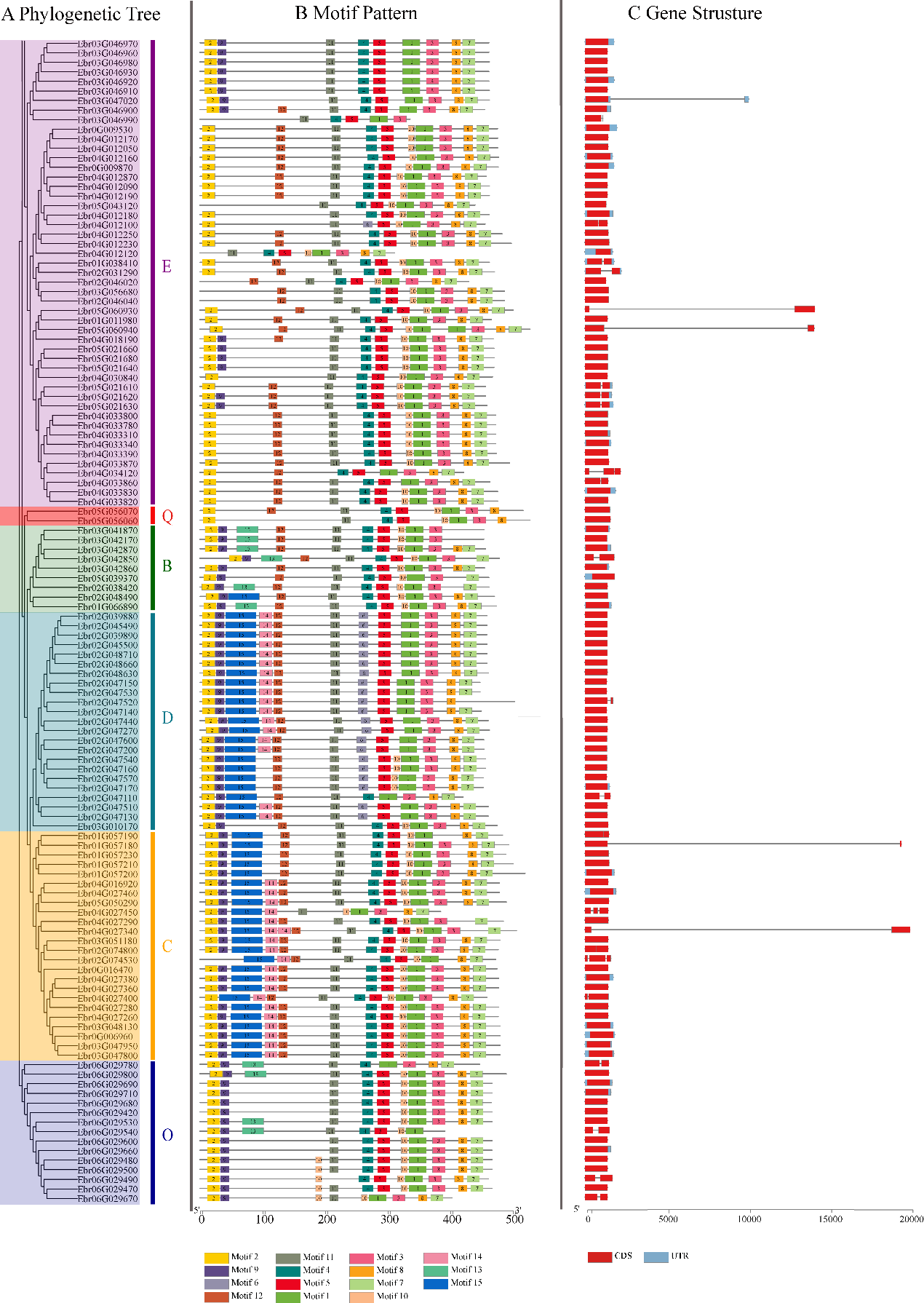


**Figure S1. Phylogenetic relationship, conservative protein motifs, and gene structures of UGTs from the groups O, Q, and B-E.** (A) The phylogenetic tree was constructed based on UGT proteins from the O, Q, and B-E groups that are indicated in different colors. (B) Conserved motifs of UGT proteins. Motifs are shown in different colored boxes. (C) Exon-intron structures of UGTs. Red boxes indicate exons, while blue boxes indicate UTR. Black lines indicate introns**.**


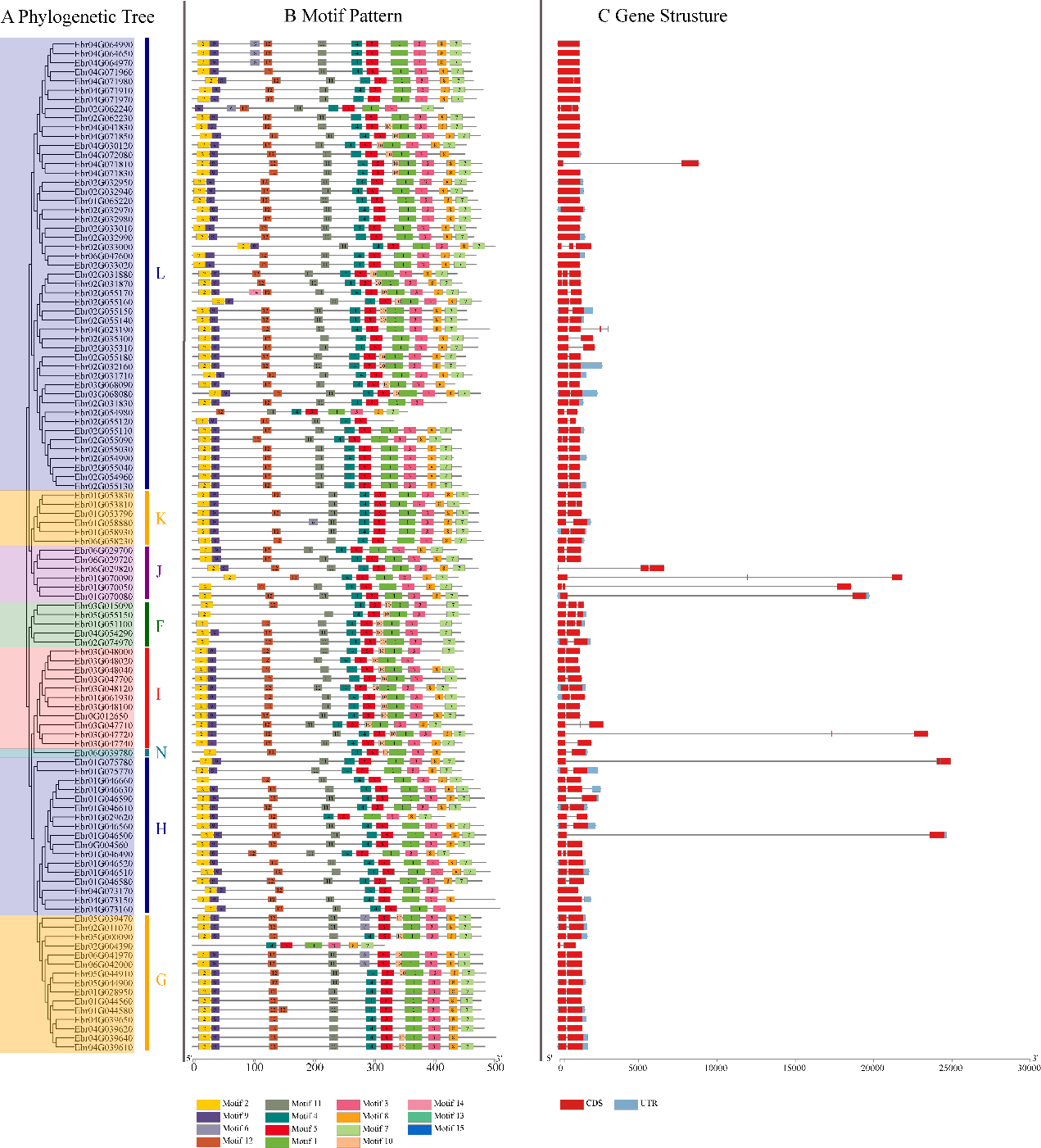


**Figure S2.** **Phylogenetic relationship, conservative motifs and gene structures of the UGTs from the groups G-L and N.** (A) The phylogenetic tree was constructed based on UGT proteins from G-L and N groups that are indicated with different colors. (B) Conserved motifs of UGT proteins. Motifs are shown in different colored boxes. (C) Exon-intron structures of UGTs. Red boxes indicate exons, and blue boxes indicate UTR, black lines indicate introns.


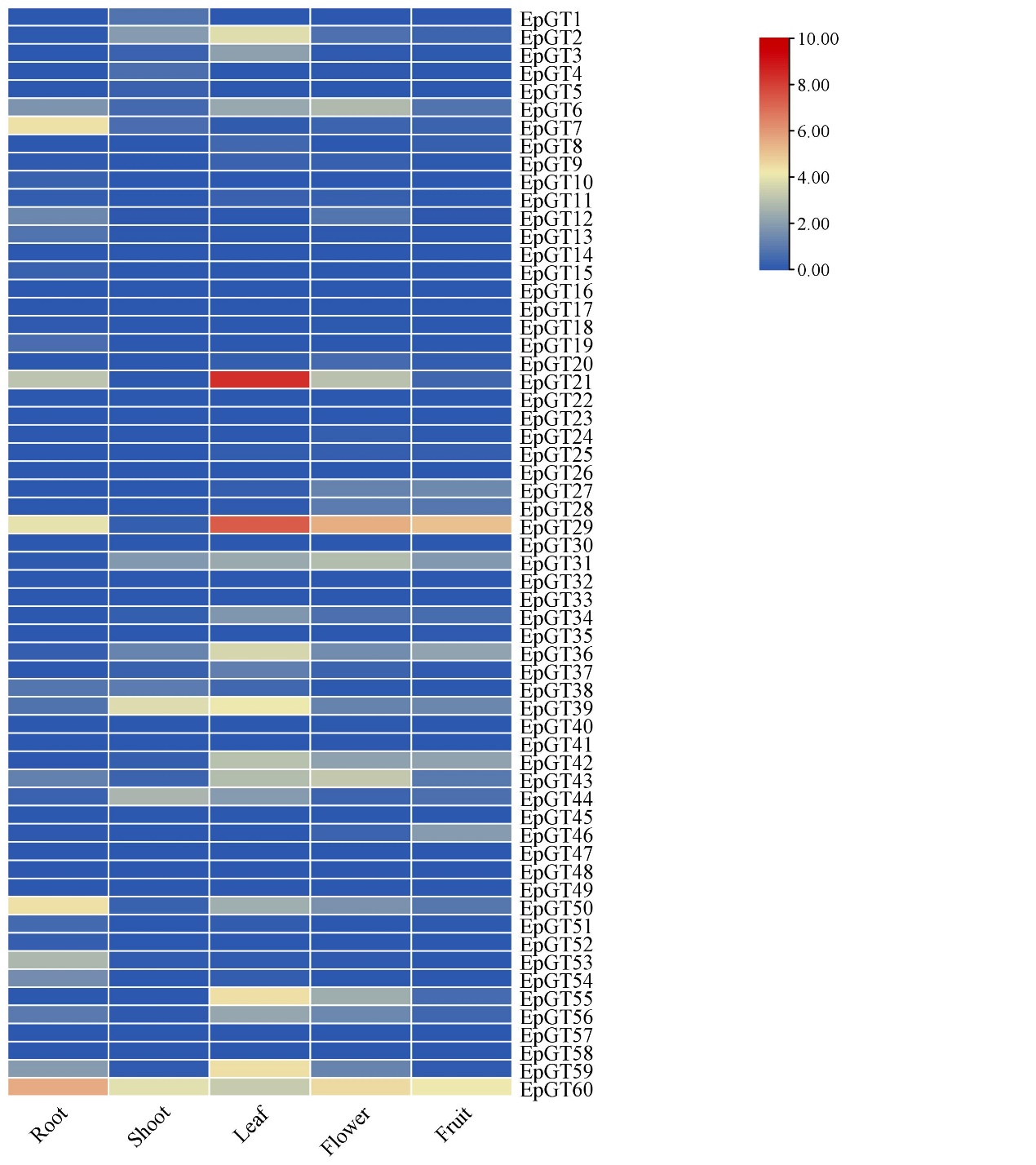


**Figure S3.** **Expression levels of *EpGT60* with UGTs from UGT79 family.** Expression levels of *EpGT60* and seleted *UGTs* from UGT79 family in various tissues by RNA-seq.


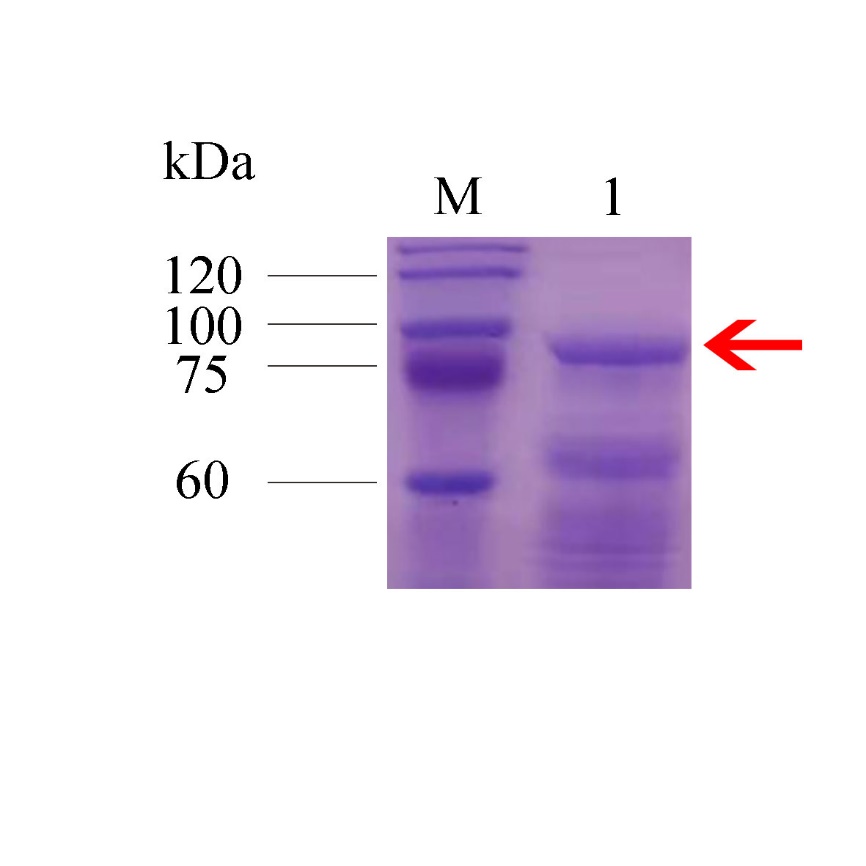


**Figure S4.** SDS-PAGE of recombinant fusion EpUGT60 proteins purified by the maltose-binding resin. Lane M: Protein Marker; Lane 1: Purified fusion EpUGT proteins. The red arrow points to the target protein.


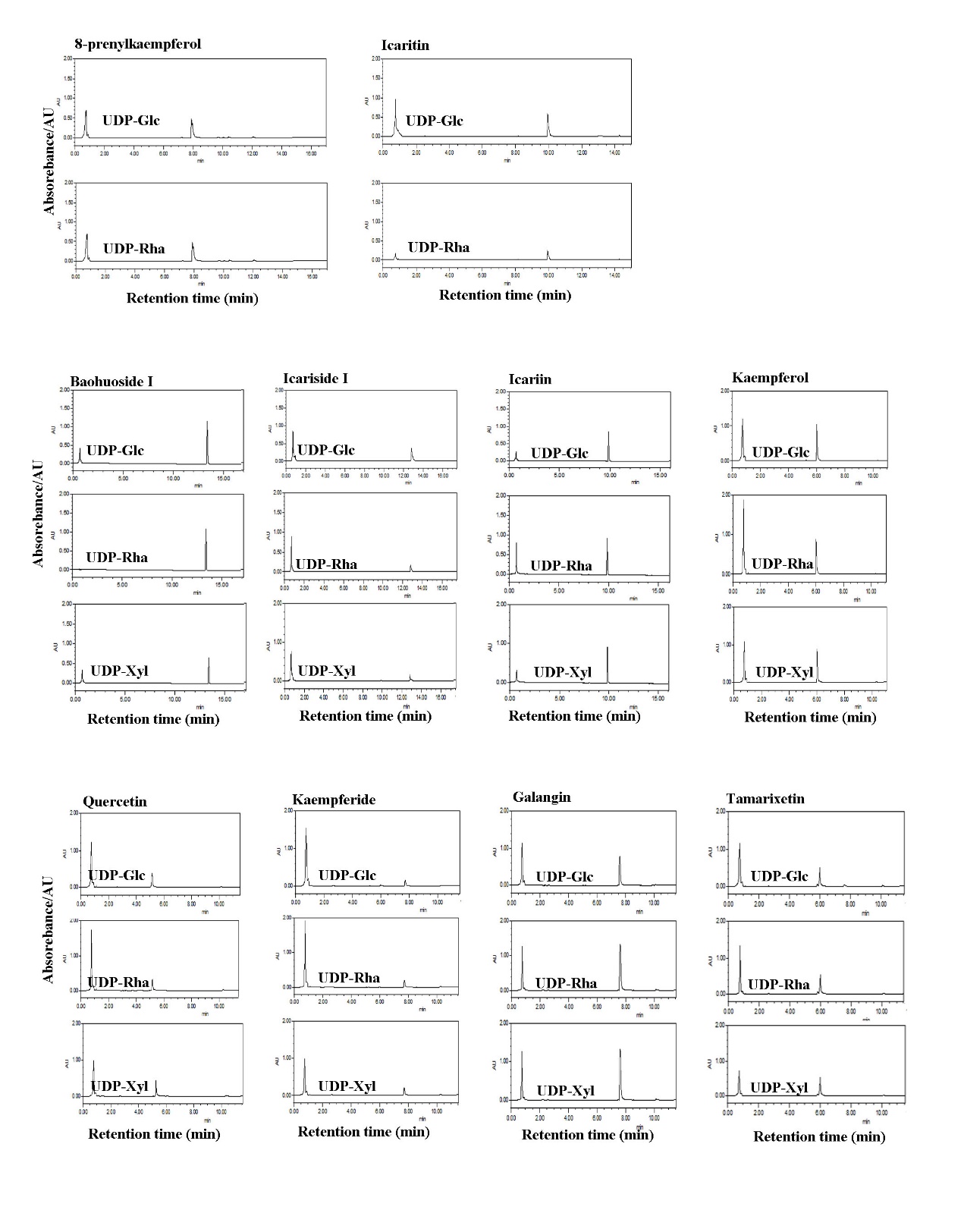

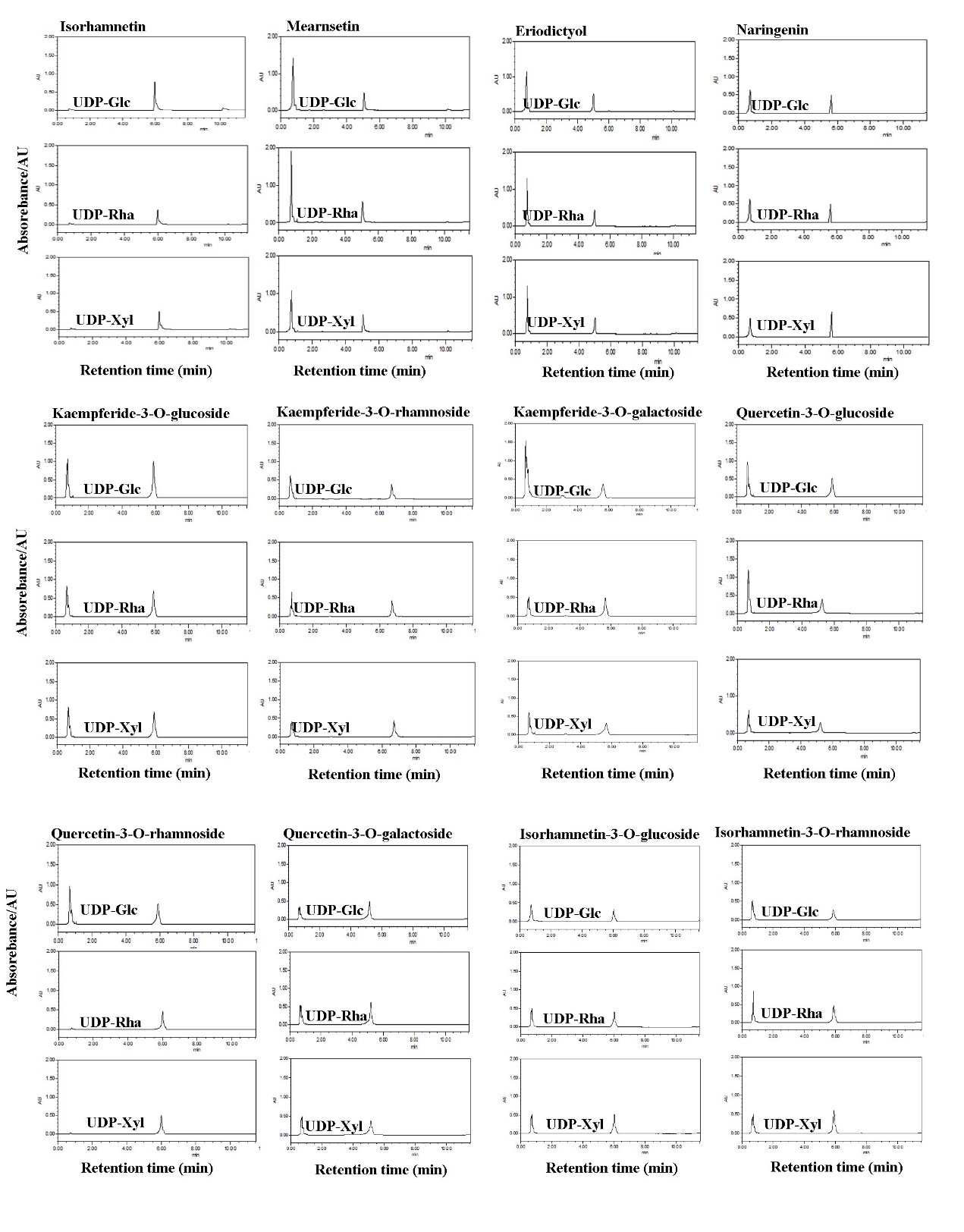


**Figure S5.** The UPLC chromatographs of reaction catalyzed by recombinant EpPT60 protein that showed no activity. Glc: glucose; Rha: rhamnose; Xyl: xylose.


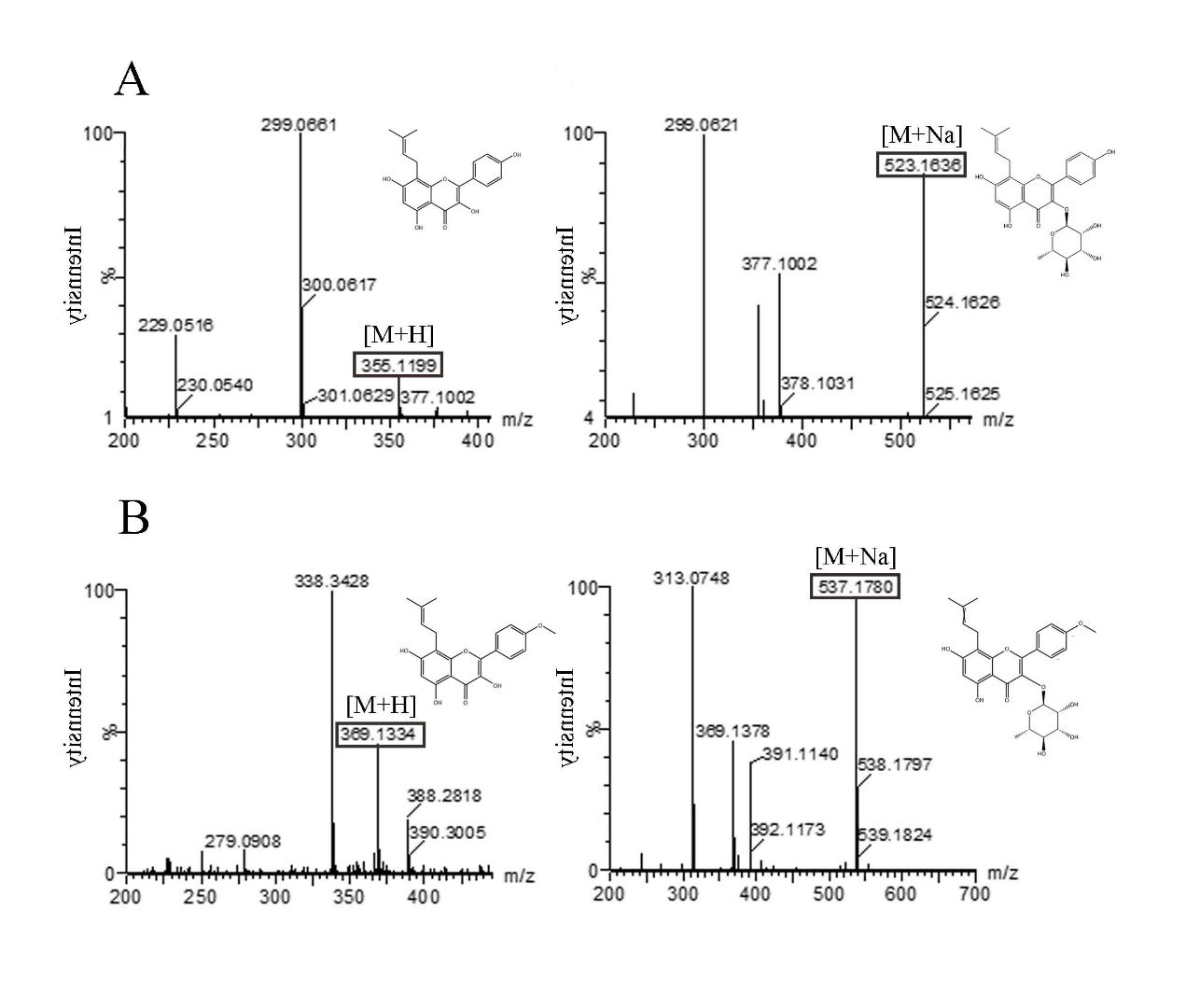


**Figure S6. Liquid chromatography mass and tandem mass spectrometry of enzymatic products of recombinant EpGT60 protein.** (A) LC-MS confirmation of 8-prenylkaempferol (left) and baohuoside II (right). (B) LC-MS confirmation of icaritin (left) and baohuoside I (right).

**
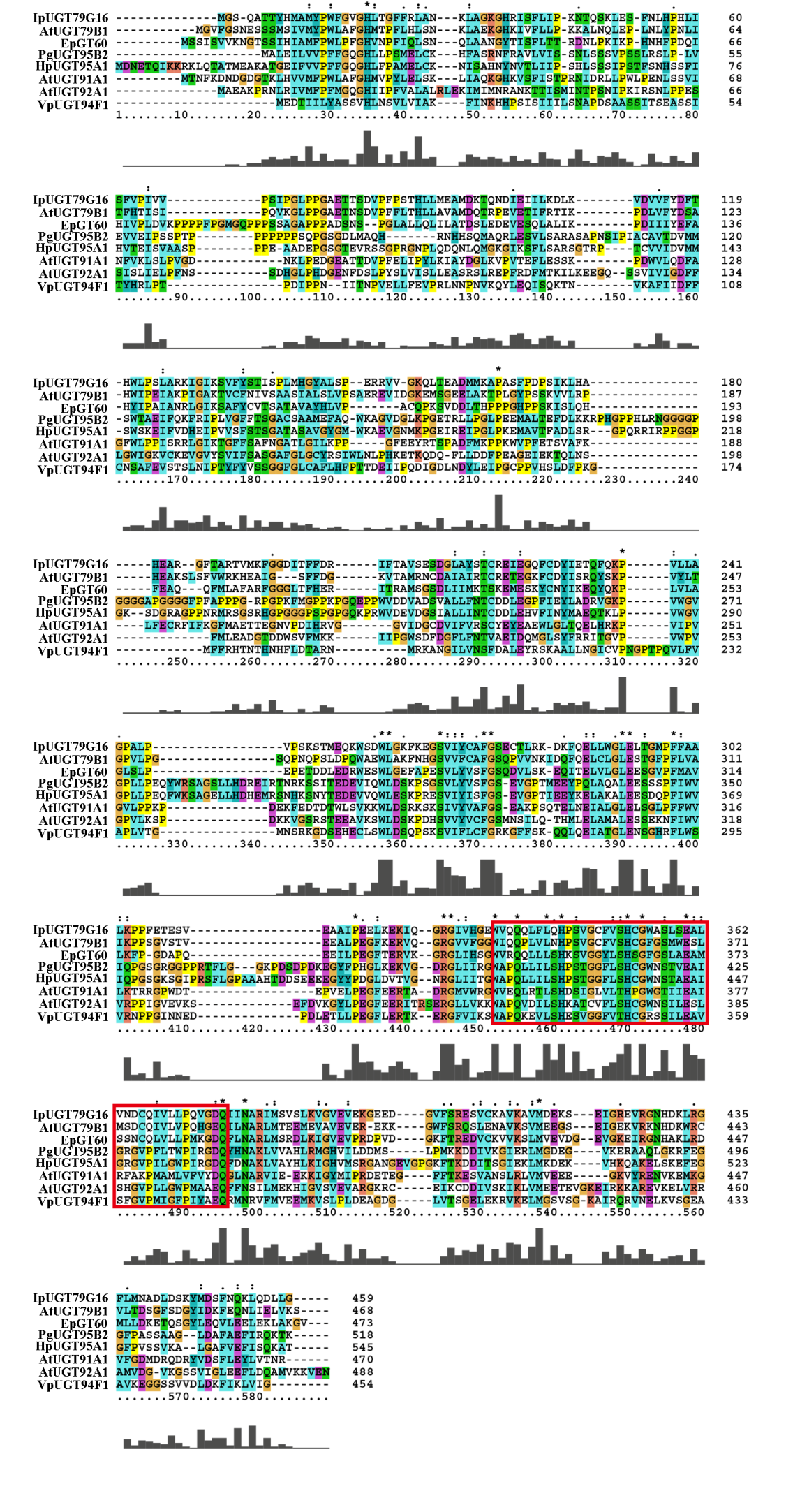
**

**Figure S7. Multiple alignment of amino acid sequences from EpGT60 and seven UGTs of representative plants.** The red box indicates the conserved region of plant secondary product glycosyltransferases (PSPG motif). Representative plant UGTs were described in Supplementary Table 2.


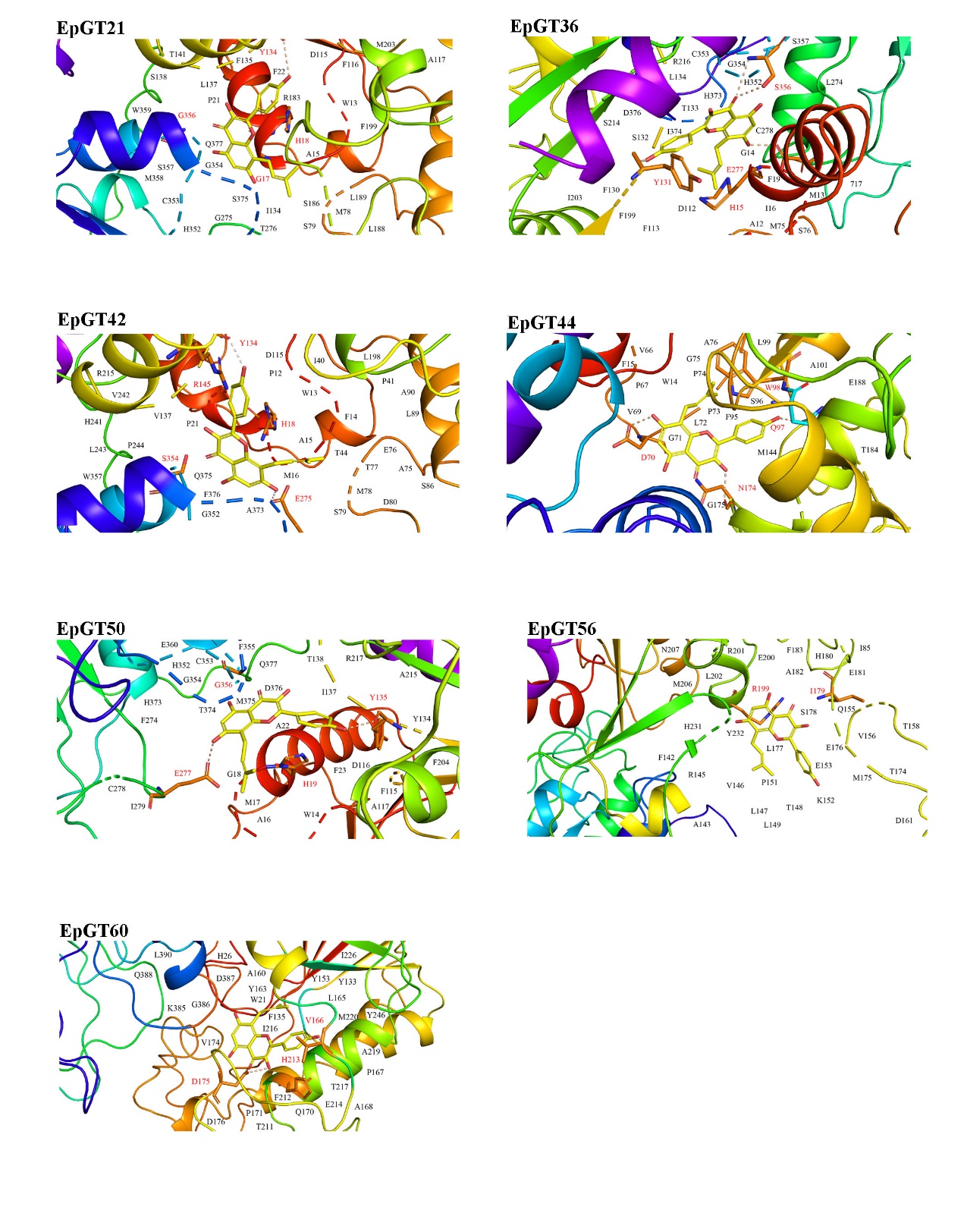


**Figure S8. Model of the EpGT21, EpGT36, EpGT42, EpGT44, EpGT50, EpGT56 and EpGT60 with 8-prenylkaempferol.**


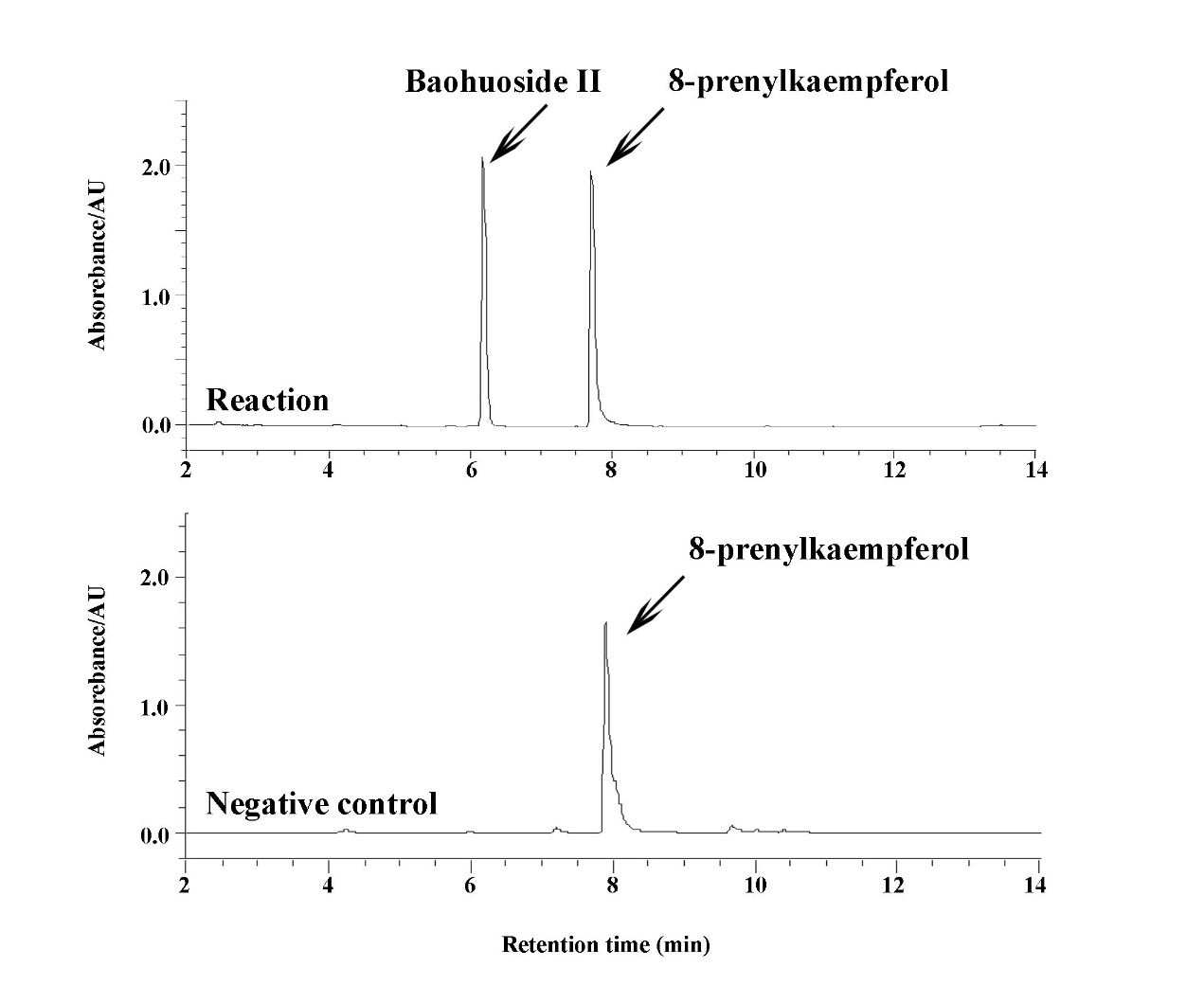


**Figure S9. The chromatographs of baohuoside II in cell culture by feeding assay with pMAL-c2X-EpGT60**


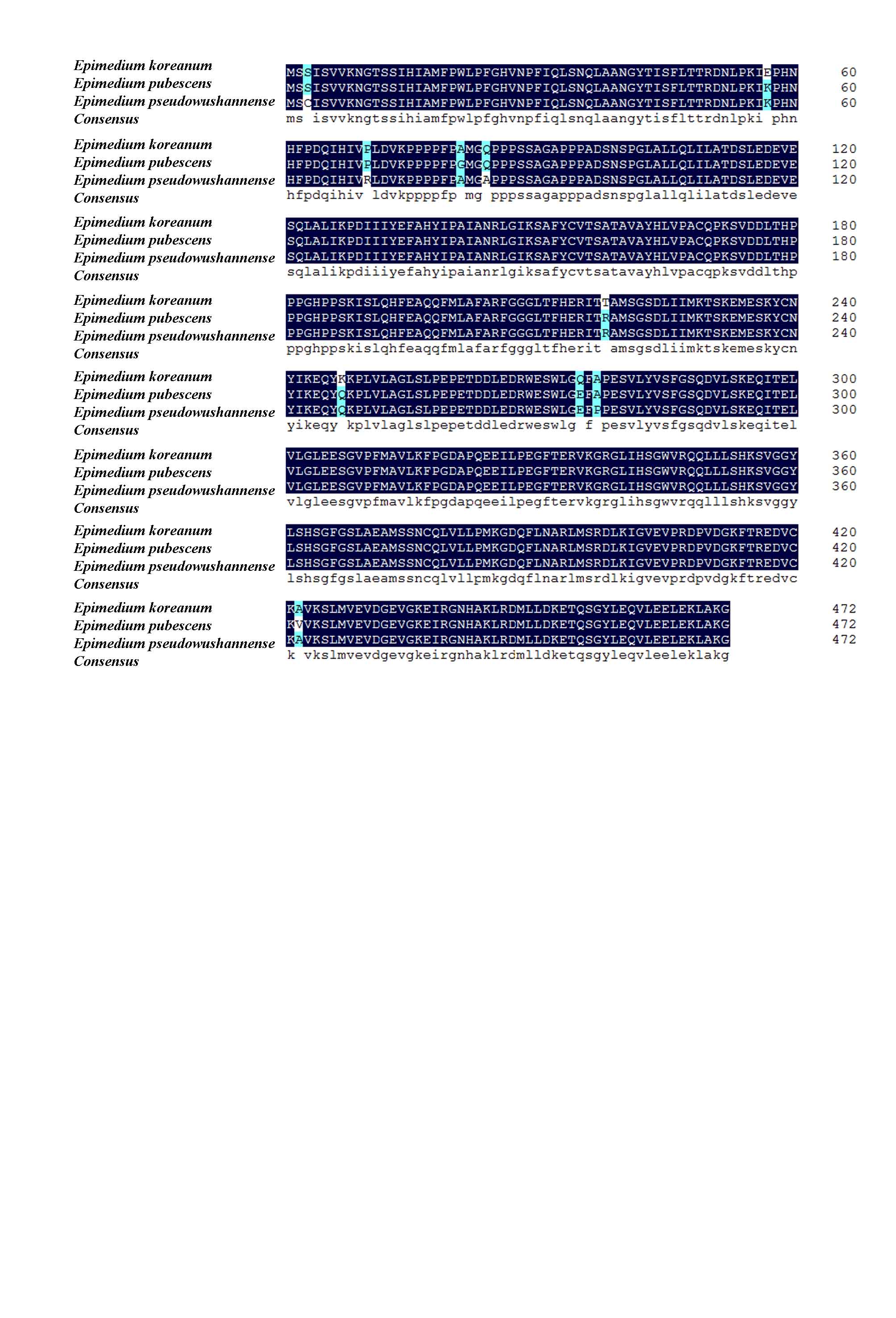


**Figure S10.** **Multiple alignment of amino acid sequences from EpGT60, EpPF3RT (accession numbers: MG264429), and EkF3URhaT (accession numbers: QTU90761.1)**.
